# Biochemical Characterization of Different Chemical Components of *Parthenium hysterophorus* and Their Therapeutic Potential against HIV-1 RT and Microbial Growth

**DOI:** 10.1155/2022/3892352

**Published:** 2022-04-28

**Authors:** Jyotsna Jaiswal, Pawan Kumar Doharey, Reetika Singh, Priyanka Tiwari, Nitika Singh, Abhishek Kumar, Vivek Kumar Gupta, Arif Jamal Siddiqui, Bechan Sharma

**Affiliations:** ^1^Department of Biochemistry, Faculty of Science, University of Allahabad, Prayagraj 211002, India; ^2^Department of Biology, College of Science, University of Hail, Hail, P.O. Box 2440, Saudi Arabia

## Abstract

*Parthenium hysterophorus* possesses certain allelochemicals responsible for their medicinal effects. The presence of oils, polyphenols, alkaloids, terpenes, pseudoguaianolides, and histamines in *P. hysterophorus* has been shown to exhibit medicinal properties. However, the systematic biomedical properties of this plant are still unexplored. The extracts of leaves, stem, and flower of *P. hysterophorus,* both at low and high temperatures (equivalent to boiling points of different solvents) were prepared. The extracts prepared in hexane, ethylacetate, methanol, and water were analyzed spectrophotometrically and colorimetrically and resolved on TLC for the presence of phytochemicals. The analyses of the free radical quenching potential of plant extracts were done by DPPH assay. The total antioxidant capacity was determined by phosphomolybdate assay and the ferric reducing antioxidant power (FRAP) assay was used to determine the reduction potential of the extracts. The spectrophotometric and qualitative analysis of plant extracts demonstrated the presence of alkaloids, terpenoids, carbohydrates, and cardiac glycosides. The occurrence of more than one Rf values for extracts determined by TLC indicated the presence of more than one phytochemical compound. The *P. hysterophorus* extracts contained strong antioxidant activity. These extracts exhibited strong antimicrobial activity against *Staphylococcus epidermis*, *Salmonela typhi*, *Neisseria gonococci* or *gonococci*, *Citrobacter*, and *Shigella flexineri.* The evaluation of the antimicrobial potential of *P. hysterophorus* extracts was done by the disc diffusion method. These extracts also showed significant inhibition against HIV-1 RT activity. The anti-HIV-1 RT activity was done using Roche Kit. The *P. hysterophorus* extracts displayed the presence of many phytochemicals with strong antioxidant, antimicrobial, and anti-HIV-1 RT properties.

## 1. Introduction

*P. hysterophorus* (L.) belongs to the family Asteraceae. The Parthenium word is derived from the Latin word ‘parthenice' [1]. The word *hysterophorus* was taken from the Greek words *hysteria* and *phoros* mean womb and bearing, respectively [2]. It is an invasive alien species which were introduced accidentally to India and spread rapidly throughout the country [3]. *P. hysterophorus* was thought to be medicinally less important and a harmful weed [4, 5].

The invasion of *Parthenium* can destroy native vegetation, local pasture, crop, and grass lands. Upon invasion, they compete for nutrients and reduce the growth of crops by releasing allelopathic chemicals in the soil and environment [6]. The destruction of the habitat of many plants including herbs, shrubs, and trees by its invasion is considered a major threat to biodiversity [7, 8].

It has been reported that *P. hysterophorus* trichomes and pollens contain toxic chemicals such as sesquiterpene lactones (SQL). A bitter glycoside parthenin is also found in it as a major sesquiterpene lactone. Some other phytotoxic constituents/allelochemicals reported so far include anisic acid, caffeic acid, chlorogenic acid, ferulic acid, fumaric acid, p-anisic acid, p-hydroxy benzoin, p-coumaric, sitosterol, and vanillic acid. In addition, some unidentified alcohols, ambrosin, hysterin, flavonoids including 6-hydroxyl kaempferol 3-0-arabinoglucoside, quercelagetin 3,7-dimethylether, and fumaric acid are also present in this plant. Among these, parthenin, hymenin, and ambrosin have been found to be highly toxic to the health of humans and animals [9].

Parthenium is a noxious weed and is said to be reportedly found in Southern and Northern America as a native species. Parthenium has been reported to inhibit the growth of other vegetation [10]. In a recent study by Ahmad et al., it is found that *P. hysterophorus* exhibits tolerance to great extent against abiotic stresses including high light intensities [11, 12].

The growth of Parthenium in agricultural lands causes about 40% loss in the yield of many crops and about 90% decrease in the forage production in India [8, 13]. The failure in the management of weed results in 40-97% loss of sorghum grain yield Ethiopia [14]. In a scientific study for the exploration of antimicrobial and anticancer activity, *P. hysterophorus* silver nanoparticles (AgNPs) were synthesized using the leaf extract of the plant [15, 16]. The synthesized AgNPs were tested against clinical pathogens viz. *Escherichia coli*, *Pseudomonas aeruginosa*, *Bacillus subtilis*, *Staphylococcus aureus*, and *Enterococcus faecalis* with the maximum zone of inhibition 18, 19, 13, 17, and 12 mm, respectively, at 80 mg/mL concentration [14, 17, 18].

However, no any systematic study has been made to explore the biomedical implications of this plant, though some sporadic reports are indicating it to be active against some microorganisms and viruses. The present work illustrates the chemical/biochemical characterization and evaluation of the antimicrobial and antiviral activities of extracts of *P. hysterophorus* prepared in different solvents at room temperature as well as at high temperature equivalent to the boiling points of respective solvents using Soxhlet apparatus.

## 2. Materials and Methods

### 2.1. Chemicals and Reagents

Mueller-Hinton agar (MHA), Luria Bertani broth, miller, and ampicillin sodium injection (SAM-500) were purchased from SRL Pvt. Ltd., HiMedia, and Saralife, respectively. The solvents, Molisch reagent, Wagner's reagent, and silica gel-coated TLC plates were obtained from Merck, Darmstadt, Germany. H_2_SO_4,_ FeCl_3,_ HCl, chloroform, bismuth nitrate, glacial acetic acid, potassium iodide, anisaldehyde, ascorbic acid, ammonium molybdate, sodium phosphate (Na_3_PO_4_), potassium ferricyanide K_3_Fe(CN)_6_, trichloroacetic acid (TCA), ferric chroride (FeCl_3_), and mercuric chloride as well as all other reagents used were of analytical and molecular grade.

### 2.2. Collection of Plant Materials and Preparation of Extracts

The leaves, stem, flower, and fruits of the *P. hysterophorus* were obtained from Prayagraj, India and adjoining areas. These plant parts were washed thoroughly using running water followed by double distilled water (ddW), shade-dried, and crushed to powder. The powder was used for extraction of phytochemicals in solvents such as hexane, ethyl acetate, methanol, and water, at room temperature as well as at high temperature (equivalent to the boiling point of the respective solvents) using Soxhlet apparatus. The boiling points for the hexane, ethyl acetate, methanol, and water are 68°C, 77.1°C, 64.7°C, and 100°C, respectively. The extracts were evaporated to dryness. The dried extracts were dissolved in the required volume of the corresponding solvents to use in different concentrations for various experiments.

### 2.3. Qualitative Assay of Phytochemicals

#### 2.3.1. Spectrophotometric Analysis of Plant Extracts

The UV-visible absorption pattern of different chemical constituents present in these plant extracts was determined by spectrophotometric analysis. Different concentrations of each of these extracts were taken in a quartz cuvette (10 mm light path, 1 mL volume) and the UV-visible spectra were monitored in the UV-visible range between 190 and 750 nm using Thermo double beam UV-Vis spectrophotometer (Scientific Spectroscan UV 2700) [18]. The absorption peaks were analyzed and matched with the absorption pattern of specific phytochemicals.

#### 2.3.2. Chemical Analysis of Plant Extracts

The qualitative chemical analysis of some plant molecules present in the extracts was carried out using the standard procedures [19, 20].

#### 2.3.3. Alkaloids

5 mg extract was dissolved in 1 mL (double distilled water) DDW. Into this, 5 drops of 1% HCl were added and steam was passed through it. To the 1 mL of this solution, 6 drops of Wagner's reagent were added. The appearance of brownish-red precipitate indicated the presence of alkaloids [20].

#### 2.3.4. Carbohydrates

5 mg extract was dissolved in 1 mL solvent (respective solvent). To it, 5 drops of Molisch reagent were added. The mixture was allowed to stand for 2-3 min. The formation of red or dull violet color indicated the presence of carbohydrates.

#### 2.3.5. Cardiac Glycosides

5 mg extract was dissolved in 2.5 mL solvent (respective solvent). To it, 2 mL of glacial acetic acid was added containing 5 drops of FeCl_3_ (5% *w*/*v*). Then, 500 *μ*L of concentrated H_2_SO_4_ was added. The formation of a brown ring indicated the presence of cardiac glycosides [20].

#### 2.3.6. Flavonoids

In 1 mL of extract containing its 5 mg, a few drops of sodium hydroxide were added. Formation of yellow color took place, which disappeared upon the addition of a few drops of 70% HCl.

#### 2.3.7. Phenols

5 mg extract was dissolved in 2 mL DDW. Upon addition of 10%FeCl_3_ (10 drops) to it, the blue-green color appeared which indicated the presence of phenols.

#### 2.3.8. Steroids

5 mg extract was dissolved in 2 mL of chloroform. To it, 2 mL H_2_So_4_ was added. The topmost layer of sulphuric acid turned red. Further, it turned into yellow color with green fluorescence showing the presence of steroids [21].

#### 2.3.9. Saponins

5 mg of extract was dissolved in 1 mL of DDW. Upon shaking the test tube, formation of persistent foam took place indicating the presence of saponins.

#### 2.3.10. Tannins

5 mg of extract was dissolved in 1 mL of DDW. It was added with 5% FeCl_3_. The appearance of black-blue precipitate indicated the presence of tannins.

#### 2.3.11. Terpenoids

In 1 mL chloroform, 5 mg of extract was dissolved followed by the addition of 10 drops of concentrated sulphuric acid. The formation of reddish-brown precipitate at the interface indicated the presence of terpenoids.

#### 2.3.12. Quinones

5 mg extract was dissolved in 1 mL solvent (respective solvent). To it, 1 mL concentrated H_2_SO_4_ was added. The formation of the red-colored complex indicated the presence of quinones.

### 2.4. DPPH Assay of Extracts at High Temperature

The DPPH radical scavenging activity assay was carried out using a standard protocol with some modifications [22]. The solution of 1,1-diphenyl-2-picrylhydrazyl (DPPH) (3.94 mg/mL; 10 mM) in methanol was prepared. 1 mL of DPPH solution was added to varying concentrations (25 *μ*g/mL, 50 *μ*g/mL, 100 *μ*g/mL, 200 *μ*g/mL, 300 *μ*g/mL, and 500 *μ*g/mL) of the extracts. The absorbance was measured at 517 nm after 30 min of incubation. Ascorbic acid (1%) was used as a standard at different concentrations (1.0 *μ*g/mL, 1.5 *μ*g/mL, 2.0 *μ*g/mL, 2.5 *μ*g/mL, and 3.0 *μ*g/mL). Simultaneously, a blank was prepared without adding extract and the absorbance was recorded. Then the absorbance was monitored in the presence of different concentrations of extracts (25 *μ*g/mL, 50 *μ*g/mL, 100 *μ*g/mL, 200 *μ*g/mL, 300 *μ*g/mL, and 500 *μ*g/mL). The low value of absorbance indicates high free radical scavenging activity. The capacity of extract to scavenge free radical was calculated by the equation:

(1) $$\begin{array}{c} \text{DPPH scavenging effect}=\left[\left(A_{0}-A_{s}\right)\times 100\right], \end{array}$$

where *A*_0_ is absorbance without adding extract and *A*_*s*_ is absorbance after addition of extract. The percentage of DPPH scavenging versus the concentration of the extract was plotted.

### 2.5. Phosphomolybdate (Total Antioxidant Capacity (TAC)) Assay

The total antioxidant capacity of different extracts of *P. hysterophorus was* determined by standard protocol [23]. For each reaction, 0.1mL of sample was added with 1 mL reagent solution (0.6M sulphuric acid, 28mM sodium phosphate, and 4mM ammonium molybdate). The test tubes were covered and incubated at 95°C for 90 min in a water bath. The sample were cooled down, and absorbance was taken at 695nm. Ascorbic acid was used as a standard. The antioxidant capacity was estimated using the following formula:

(2) $$\begin{array}{c} \%\text{TAC inhibition}=\left(\frac{\text{Abs sample}-\text{Abs control}}{\text{Abs sample}}\right)\times 100, \end{array}$$

Abs sample = Absorbance of sample + reagent solution.

Abs control is equal to the absorbance of reagent solution and solvent of plant extract.

### 2.6. The Ferric Reducing Antioxidant Power (FRAP) Assay

The FRAP of different extracts of *P. hysterophorus* was tested using the potassium ferricyanide-ferric chloride method with some modification [24, 25]. For each reaction, 1 mL plant extract (5 mg/mL) was added to 2.5 mL of phosphate buffer (0.2 M, pH 6.6) and 2.5 mL (1%) potassium ferricyanide (K_3_Fe(CN)_6_). The tubes were incubated for 20 min at 50°C in a water bath. 2.5 mL of trichloroacetic acid (TCA) (10%) was added in to it. After mixing, samples were centrifuged for 10 min at 3000xg, and the supernatant was collected and used for further processes. The supernatant (2.5 mL) was added with deionized water (2.5 mL) and 0.5 mL of 1% FeCl_3_ solution. The reactants were gently mixed, and the mixture was allowed to stand for 30 min at RT. The optical absorbance of mixture was recorded at 700 nm. Ascorbic acid was used as a standard. The values were presented as redox potential equivalent to Vit. C (*μ*g/mg extract).

### 2.7. Thin Layer Chromatography (TLC)

Silica gel-coated TLC plates (Merck, Darmstadt, Germany) were used for phytochemical analysis using various extracts of *P. hysterophorus* leaves prepared in water and ethyl acetate at room temperature (RT). The *P. hysterophorus* flowers extracts were prepared in methanol at high temperature (HT) and at RT in ethyl acetate and used on TLC [21]. The plates were dried and activated by heating at 110°C for 60 min. The extracts were dissolved in respective solvents. The extracts were applied 1.5 cm above from the bottom using glass capillary tubes. The solvent system acting as a mobile phase was allowed to run up to 7 cm from the point of application. The spots were visualized by exposure of plates to UV light and also by treatment with different developing reagents to ascertain the presence of various phytochemicals in the extracts. The retardation factor (Rf value) for each spot was calculated using the formula Rf = distance travelled by solute/distance travelled by solvent [26].

### 2.8. Evaluation of the Anti-HIV-1 RT Potential of the Plant Extracts

The assessment of the anti-HIV-1 RT potential of different plant extracts was done colorimetrically by using Reverse Transcriptase Assay Colorimetric Kit (Roche, USA). The experiments were performed according to the instruction given in the user manual. Briefly, the experiments were performed by adding 20 *μ*L of HIV-1 RT solution, 20 *μ*L plant extract, and 20 *μ*L of reaction mixture containing a hybrid of template (poly rA) and oligo dT (primer), dTTP, Mg^2+^, and buffer. One set of reaction was carried out without plant extract. The reaction mixture was incubated at 37°C for 1 h. The whole reaction mixture was transferred into 96-well plate. This system was left for incubation at 37°C for 1 h. The solution was removed completely, and the plate was washed 5 times for 30 seconds each time with washing buffer. In the next step, 200 *μ*L of antidigoxygenin- (DIG-) peroxidase (POD) (working solution) was added. It was again incubated at 37°C for 1 h. After incubation, the wells were washed 5 times in 30 seconds with washing buffer. In the further step, 200 *μ*L of ABTS substrate solution was added per well and incubated at 25°C for 30 min. In the final step, OD was measured at 405 nm (reference *λ* = 490 nm) in an ELISA plate reader.

### 2.9. Antimicrobial Activity

#### 2.9.1. Preparation of Antibiotic Stock Solution

5 mg/L of stock solution was prepared with ampicillin. 5 *μ*L of it was used in each standard well as a positive control.

#### 2.9.2. Culture Media

The media employed for the study were Luria broth and MHA media. The culture media were autoclaved at 121°C for 30 min.

#### 2.9.3. Sterilization of Material

The loop, petri dishes, pipettes, pipette tips, forceps, and disc were packed into autoclave bags appropriately and sterilized in the hot air oven at 170°C for 1 h at each experiment.

#### 2.9.4. Test Microorganisms

Five different clinical pathogenic bacterial species viz. *Salmonella typhi*, *Neisseria gonorrhoeae* (*N. gonorrhoeae*), *Citrobacter*, *Shigella flexneri*, and *Staphylococcus epidermidis* were used. The bacterial species were incubated in Luria broth media for 2 h using an incubator shaker at 37°C and 110 rpm.

#### 2.9.5. Antibacterial Activity

The bacterial strains were seeded in petri plates containing 20 mL solidified MHA (1 mg/mL) plants extract solution prepared in DDW. The antimicrobial activity of different concentrations of extracts was assayed using disc diffusion method (6 mm sterilized disc) placed on solidified media using sterile forceps. The plant extracts in 100, 200, 300, and 500 *μ*g in 5 *μ*L volume each quantities were added to the disc. Ampicillin (5 *μ*L, 25 *μ*g) and DDW (5 *μ*L) were used as positive and negative controls, respectively. The plates were incubated at 37°C for 24 h. The antimicrobial compounds found in the extract were allowed to diffuse into the medium and interact with the test organisms in the freshly seeded plate. The diameter of the zone of inhibitions was measured in millimeters (mm) after 24 h of incubation.

#### 2.9.6. Statistical Analysis

All experiments were independently performed in triplicate. The data were expressed as mean ± SE of three replicates, and values were analyzed statistically.

## 3. Results

### 3.1. Spectrophotometric Profiles of the Phytochemicals from P. hysterophorus Extracts Prepared at High Temperature

The extracts from leaves, stem, and flowers of *P. hysterophoru*s prepared at high temperature equivalent to the boiling point of the solvents (hexane, ethyl acetate, methanol, and H_2_O) were analyzed spectrophotometrically towards detection of the abilities of different chemical ingredients present in the extracts to absorb light in the visible and ultraviolet regions across a wide range of wavelengths, i.e., 190-700 nm. The results shown in Supplementary Figure 1 represent the appearance of various absorption peaks due to the presence of different chemical ingredients in the extracts from leaves, stem, and flowers of *P. hysterophorus*. When these peaks were compared with the standard absorption peaks reported, it was found that the presence of major absorption peaks in the range of 402, 648, and 655 nm represented the presence of flavonoids. All the *P. hysterophorus* extracts were recorded to show pheophytin A (229, 340, 410, and 669 nm) except aqueous extracts of stem and flower. Additionally, the leaf, stem, and flower extracts of the plant displayed the presence of unsaturated carbonyl compounds (201, 203 nm) in the aqueous extract. The methanolic extracts of the flowers of *P. hysterophorus* prepared at high temperature shows the presence of unsaturated carbonyl compounds; however, the aqueous extracts of all the parts of *P. hysterophorus* indicated the presence of unsaturated carbonyl compounds in the extracts prepared at high temperatures. The presence of unsaturated carbonyl compounds appeared only when a low concentration (250 *μ*g/mL) of the extract was used for spectrophotometric analysis (Supplementary Table 1) [27, 28].

### 3.2. Spectrophotometric Profiles of the Phytochemicals from P. hysterophorus Extracts Prepared at Low Temperature (24 ± 2°C)

The extracts from leaves, stem, and flowers of *P. hysterophoru*s prepared at low temperature equivalent to room temperature, i.e., 24 ± 2**°**C in different solvents (hexane, ethyl acetate, methanol, and H_2_O) were analyzed spectrophotometrically towards detection of the abilities of different chemical ingredients present in the extracts to absorb light in the ultraviolet and visible regions across a wide range of wavelengths, i.e., 190-700 nm. The results shown in Supplementary Figure 1 represent the appearance of various absorption peaks due to the presence of different chemical ingredients in the extracts from leaves, stem, and flowers of *P. hysterophorus*. When these peaks were compared with the standard absorption peaks reported, it was found that the presence of major absorption peaks in the range of 380, 410, 533, and 669 nm represented the presence of flavonoids and unsaturated carbonyl compounds. All the *P. hysterophorus* extracts were recorded to exhibit the presence of pheophytin A showing absorbance at the wavelengths 380, 410, 533, and 669 nm.

The pattern of appearance of unsaturated carbonyl compounds was the same in the methanolic extracts of flowers of *P. hysterophorus* prepared at low temperature. However, the aqueous extracts of all the parts of *P. hysterophorus* indicated the presence of unsaturated carbonyl compounds in the extracts prepared at low temperature. The presence of unsaturated carbonyl compounds appeared only when low concentration (250 *μ*g/mL) of the extract was used for spectrophotometric analysis (Supplementary Table 2).

### 3.3. Chemical Characterization of Different Plant Extracts

The presence of plant-based molecules in the extracts from different parts of the plant, *P. hysterophorus,* prepared at high and low temperatures were chemically analyzed to have their qualitative estimate.

#### 3.3.1. Qualitative Analysis of Phytochemicals Present in Different Extracts of P. hysterophorus Prepared at Low Temperature

The results presented in Table 1 indicated the presence of maximum amount of terpenoids in all the extracts prepared from leaves, stem, and flower in all the solvents tested at room temperature followed by other molecules. Saponins and tannins were found to be absent in the extracts of the leaves, stem, and flowers prepared in hexane and ethyl acetate at low temperature. Flavonoids were found absent in hexane and methanolic extracts of leaves, stem, and flowers while present in extracts of leaves and stem prepared in ethyl acetate and aqueous medium at low amount. The results presented in Table 1 indicated that the steroids and phenols were present in all parts of the plant as well as in all the solvents (hexane, eythylacetate, methanol, and H_2_O) tested in small amounts. Quinones were found to be present in the extracts prepared in hexane, ethyl acetate, and H_2_O from all the parts of the plant. However, the methanolic extracts of stem and flowers showed the presence of quinones excepting the leaves which showed its absence. Cardiac glycosides were absent in methanolic extracts of stem and flowers. It was also not detected in the aqueous extracts of all parts of *P. hysterophorus* while it was present in rest of the other extracts of different parts of the plant. Carbohydrates were present in all parts of the plant prepared in methanol and ethyl acetate. The extract of flowers of *P. hysterophorus* prepared in hexane and H_2_O registered the presence of carbohydrates. However, carbohydrates were not detected in the extracts of leaves and stem prepared in hexane and H_2_O prepared at low temperature.

Alkaloids were found to be present in high concentrations of aqueous extracts of leaves, stem, and flowers of *P. hysterophorus* prepared at room temperature. Alkaloids were also found to be present in methanolic extracts of leaves and flowers of *P. hysterophorus*.

The methanolic extract of the stem did not show the presence of alkaloids, and the extracts of leaves and flowers prepared in ethyl acetate did not show the presence of alkaloids. The ethyl acetate extract of stem indicated the presence of substantial amount of alkaloids. The extracts of stem and flowers prepared in hexane did not show presence of alkaloids; however, the leaves indicated the presence of alkaloids in its extract prepared in hexane. The results are indicated in Table 1.

#### 3.3.2. Qualitative Analysis of Phytochemicals Present in Different Extracts of P. hysterophorus Prepared at High Temperature

In the presence of plant-based molecules in the extracts from leaves of the plant, *P. hysterophorus* prepared at high temperature was chemically analyzed to assess their qualitative estimate. The results presented in Table 2 indicated the presence of varying amounts of phytochemicals in all the extracts prepared in different solvents at high temperature.

The results presented in Table 2 indicated the absence of flavonoids, saponins, and tannins in the extracts of leaves, stem, and flowers prepared in hexane. These compounds were also not detected in the ethyl acetate extracts of leaves and stem but were present in aqueous extract of leaves. The methanolic extracts of leaves and stem reported the presence of a small amount of saponins.

Terpenoids, steroids, phenols, and quinones were present in the extracts of leaves, stem, and flowers prepared in hexane and ethyl acetate. The methanolic extract of leaves and stem showed the presence of only terpenoids, phenols, and steroids were not detected in them. However, methanolic and aqueous extracts of flowers indicated the presence of these molecules. The alkaloids were found to be absent in the extracts of leaves prepared in hexane and ethylacetate. The methanolic and aqueous extracts of leaves, stem, and flowers show the presence of a substantial amount of alkaloids.

The cardiac glycosides and carbohydrates were present in the extracts of leaves, stem, and flowers when extracted in hexane, ethyl acetate, and methanol. The presence of these molecules was not detected in the aqueous extracts of flowers. The cardiac glycosides were also absent in the methanolic extract of the stem. The results are shown in Table 2.

### 3.4. Evaluation of Inhibitory Potential of the P. hysterophorus Extracts against HIV-1 RT Activity

The effect of different extracts of *P. hysterophorus* was evaluated against the activity of HIV-1 RT as described in Materials and Methods. The results demonstrated in Table 3 on extracts of *P*. *hysterophorus* leaves prepared in the aqueous medium at room temperature showed maximum inhibitory potential against the activity of HIV-1 RT with IC value of 0.081 mg/mL followed by the extract prepared in ethyl acetate (0.34 mg/mL). The extracts of *P*. *hysterophorus* leaves prepared in hexane at room temperature showed inhibitory activity at (0.65 mg/mL), while *P. hysterophorus* leaves prepared in methanol at room temperature did not show any anti-HIV-1 RT activity. In the case of a high-temperature extraction of the compounds, the extract of *P*. *hysterophorus* leaves prepared at high temperature in hexane showed the highest inhibitory activity (IC_50_ value 0.44 mg/mL) against HIV-1 RT followed by *P. hysterophorus* leaves prepared in ethyl acetate at high temperature (IC_50_ value 0.55 mg/mL) (Table 3).

The results shown in Table 4 indicated that the extracts of flowers of *P. hysterophorus* prepared in ethyl acetate at high temperature showed strong inhibition potential against HIV-1 RT with the IC_50_ value of 0.31 mg/mL. Other extracts of flowers prepared at low temperature (room temperature) in water and methanol did not show any inhibitory activity against HIV-1 RT. The extract of *P. hysterophorus* flowers prepared in ethyl acetate at high temperature showed the highest inhibition against HIV-1 RT with the IC_50_ value being 0.40 mg/mL followed by extract methanol in (IC_50_ value, 0.47 mg/mL) (Table 4).

### 3.5. Analysis of Phytochemicals Highly Present in Different Plant Extracts by Thin-Layer Chromatography (TLC)

#### 3.5.1. Phytochemical Analysis of Extract of *P. hysterophorus* Flowers in Ethyl Acetate Medium/Solvent at Room Temperature

The presence of various phytochemicals in the plant extracts of *P. hysterophorus* flowers prepared at room temperature in ethyl acetate was analyzed by using the thin layer chromatography (TLC) technique. The samples were resolved and analyzed using TLC plates as indicated in Materials and Methods. Specific reagents were sprayed on TLC plates to obtain colored spots corresponding to different phytochemicals present in the extracts.

**Figure 1:**
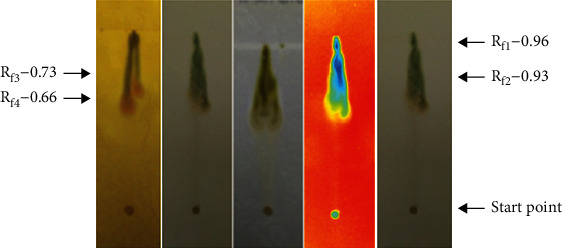
Phytochemical analysis of the ethyl acetate extract of *P*. *hysterophorus* flower prepared at room temperature. (a) Detection of alkaloids and primary and secondary amines using Dragendorff reagent. (b) Detection of natural products using anisaldehyde-sulphuric acid reagent. (c) Detection of alkaloids using Mayer's reagent. (d) Detection of compounds using UV light. (e) Detection of compounds using visible light.

Figure 1(a) indicates the treatment of TLC plate with Dragendorff reagent to detect the flavonoids as well as primary and secondary amines. The solvent system used for the detection was methanol, ethyl acetate, hexane, acetic acid in the ratio of 2: 7 : 1 : 0.5. Their Rf values were calculated using the formula, i.e., the ratio of distance traveled by solute and the solvent. The Rf values were found to be 0.66, 0.73, and 0.93. The flavonoids being the most polar compounds showed the lower Rf values as against the amines which were relatively less polar and traveled higher distance.

Figure 1(b) indicates the TLC plate treated with anisaldehyde-sulphuric acid reagent to develop colored complexes of the phytochemicals such as antioxidants, steroids, prostaglandins, carbohydrates, phenols, glycosides, saponins, myotoxin, essential oil, and terpenes with their Rf values equal to 0.66, 0.93 and 0.96. Among these, the carbohydrates, glycosides, phenols, and many antioxidants were polar in nature, and hence, they might have lower Rf values than those compounds, which were nonpolar such as steroids, prostaglandins, essential oils, and terpenes. The saponins and mycotoxins may have intermediate Rf values as some of them have both polar and nonpolar characteristics.

In order to resolve and detect the presence of flavonoids in the extract of flowers of *P. hysterophorus* prepared in ethyl acetate was done by spraying Mayer's reagent on the TLC plate. The results indicated the appearance of colored spots (Figure 1(c)). The Rf values of different flavonoids present in this extract were calculated, and the values were 0.66, 0.73, 0.93, and 0.96.

The presence of the photoactive compounds in the extract of flower of *P. hysterophorus* prepared/extracted in ethyl acetate was detected by applying and resolving the extract in the TLC plate (using the reagent, Dragendorff reagent, anisaldehyde-sulphuric acid reagent, and Mayer's reagent) as mentioned in Materials and Methods. The dried TLC plates were exposed to UV radiation, and the spots were identified and their corresponding Rf values were calculated to be 0.66, 0.93, and 0.96 (Figure 1(d)). The presence of phytochemicals in the ethyl acetate extract of *P. hysterophorus* flowers were detected by resolving the extract on TLC plate and observing it by necked eyes. A total of three spots were observed with Rf values of 0.66, 0.93, and 0.96 representing the presence of different chemical ingredients as shown in Figure 1(e).

#### 3.5.2. Phytochemical Analysis of Extract of *P. hysterophorus* Leaves in Aqueous Medium at Room Temperature

**Figure 2:**
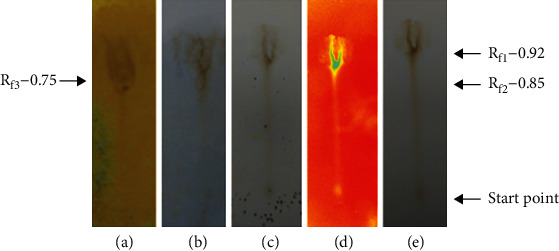
Phytochemical analysis of the aqueous extract of *P. hysterophorus* leaves prepared. at room temperature. (a) Detection of alkaloids and primary and secondary amines using Dragendroff's reagent. (b) Detection of naturalproducts using anisaldehyde-sulphuric acid reagent. (c) Detection of alkaloids using Mayer's reagent. (d) Detection of compounds using UV light. (e) Detection of compounds using visible light.

The presence of various phytochemicals in the extracts of *P. hysterophorus* leaves prepared at room temperature in an aqueous medium was analyzed by using TLC technique. The results are shown in Figure 2.

Figure 2(a) indicates the presence of flavonoids and their derivates as well as primary and secondary amines for the *P. hysterophorus* leaf prepared at room temperature in aqueous medium, in the TLC plates on the treatment with Dragendorff reagent. The solvent system used for the detection was ethyl acetate, formaldehyde, water in the ratio of 6.5 : 2 : 1.5. Their Rf values were calculated and found to be 0.75 and 0.92.

Figure 2(b) shows the TLC plate treated with anisaldehyde-sulphuric acid reagent to develop colored complexes of the phytochemicals such as antioxidants, steroids, prostaglandins, carbohydrates, phenols, glycosides, saponins, myotoxin, essential oil, and terpenes and their Rf values were detected as 0.75, 0.85, and 0.92. The polar compounds carbohydrates, glycosides, phenols, and many antioxidants have lower Rf values as compared to those compounds which are nonpolar such as steroids, prostaglandins, essential oils, and terpenes. Other compounds like saponins and myotoxin, may have intermediate Rf values because some of them have both polar and nonpolar characteristics. Figure 2(c) indicates the presence of flavonoids in the extract of *P. hysterophorus* leaf prepared at room temperature in aqueous medium by spraying Mayer's reagent on the TLC plate. The Rf value for the flavonoids was observed as 0.92. The photoactive compounds were detected by exposing the dried TLC plates in the UV radiation, and the spots were identified and their corresponding Rf values were calculated to be 0.85 and 0.92. The results were shown in Figure 2(d). The presence of phytochemicals as observed in the visible range were detected by resolving the extract on TLC plate and observing it by necked eyes. A single spot was visible showing the presence of different chemical compounds with a corresponding Rf value of 0.92 (Figure 2(e)).

#### 3.5.3. Phytochemical Analysis of Extract of P. hysterophorus Leaves in Ethyl Acetate Medium at Room Temperature

**Figure 3:**
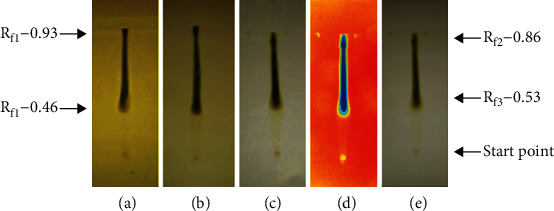
Phytochemical analysis of the ethyl acetate extract of *P*. *hysterophorus* leaves prepared at room temperature. (a) Detection of alkaloids and primary and secondary amines using Dragendorff's reagent. (b) Detection of natural products using anisaldehyde-sulphuric acid reagent. (c) Detection of alkaloids using Mayer's reagent. (d) Detection of compounds using UV light. (e) Detection of compounds using visible light.

The presence of various phytochemicals in the plant extracts of *P. hysterophorus* leaves prepared at room temperature in ethyl acetate medium was analyzed by TLC technique.  Figure 3(a) indicated the treatment of TLC plate with Dragendorff reagent to detect the flavonoids as well as primary and secondary amines. The solvent system used for the detection was methanol, ethyl acetate, hexane, and acetic acid in the ratio of 2: 7 : 1 : 0.5 as described in Materials and Methods. The Rf values were found to be 0.86 and 0.93 for different fractions of the extracts. Flavonoids are the most polar compounds that showed the lower Rf values whereas amines are relatively less polar and travelled a higher distance.

Figure 3(b) indicated the TLC plate treated with anisaldehyde-sulphuric acid reagent to develop colored complexes of the phytochemicals such as antioxidants, steroids, prostaglandins, carbohydrates, phenols, glycosides, saponins, myotoxin, essential oil, and terpenes with their Rf values equal to 0.86 and 0.93. The polar compounds such as carbohydrates, glycosides, phenols, and many antioxidants may have lower Rf values than nonpolar compounds such as steroids, prostaglandins, essentialoils, and terpenes. Some compounds such as saponins and myotoxin may have intermediate Rf values because some of them have both polar and nonpolar characteristics.

In order to resolve and detect the presence of flavonoids in the extract of leaves of *P. hysterophorus* prepared in ethyl acetate, the TLC plate was sprayed with Mayer's reagent. The results indicated the appearance of colored spots (Figure 3(c)). The Rf values of different flavonoids present in this extract were calculated, and the values were found to be 0.53 and 0.86. The photoactive compounds present in the extract *P. hysterophorus* leaves prepared in ethyl acetate were detected by exposing the dried TLC plates to UV radiation. The corresponding Rf values were computed for the corresponding spots and the values were found to be 0.53 and 0.86 (Figure 3(d)). The same plate was also observed by naked eyes, and the spots were identified with the Rf values of 0.53 and 0.86 representing the presence of different chemical ingredients as shown in Figure 3(e) (Table 5).

#### 3.5.4. Phytochemical Analysis of Methanolic Extract of P. hysterophorus Flowers at High Temperature

**Figure 4:**
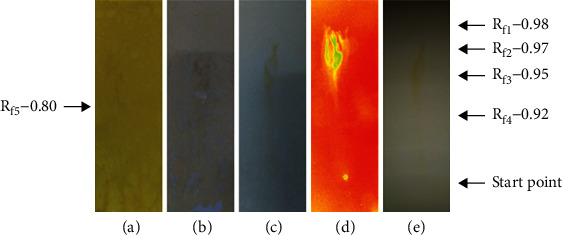
Phytochemical analysis of the methanolic extract of *P. hysterophorus* flower prepared at high temperature. (a) Detection of alkaloids and primary and secondary amines using Dragendroff's reagent. (b) Detection of phytochemicals using anisaldehyde-sulphuric acid reagent. (c) Detection of alkaloids using Mayer's reagent. (d) Detection of photoactive compounds using UV light. (e) Detection of compounds using visible light.

The presence of a variety of phytochemicals in the methanolic extract of *P. hysterophorus* flower prepared at high temperature was analyzed by using TLC technique.  Figure 4(a) indicated the treatment of TLC plate with Dragendorff reagent to detect the flavonoids as well as primary and secondary amines. The solvent system used for the detection was toluene, acetone, acetic acid in the ratio of 9 : 1 : 0.5. The Rf values for the compounds resolved were calculated by using the formula mentioned above. The Rf values were found to be 0.80 and 0.92. Amines had higher Rf values because they are less polar and traveled higher distances whereas flavonoids being the most polar compounds showed the lower Rf values and traveled short distances in comparison to amines. Another set of TLC was performed to resolve the extract. The TLC plate was sprayed with anisaldehyde-sulphuric acid reagent to detect the presence of phytochemicals such as antioxidants, steroids, prostaglandins, carbohydrates, phenols, glycosides, saponins, myotoxin, essential oil, and terpenes (Figure 4(b)). A TLC plate showed only one thick spot with Rf values equal to 0.95.

Among these compounds the polar compounds carbohydrates, glycosides, phenols, and many antioxidants are may have lower Rf values in contrast to the compounds such as steroids, prostaglandins, essential oils, and terpenes which are nonpolar in nature. The compounds which may have intermediate Rf values are saponins and myotoxin because some of them have both polar and nonpolar characteristics (Figure 4(b)).

In the presence of different flavonoids in this extract, the sample was resolved on the TLC plate and sprayed with Mayer's reagent as described in Materials and Methods. The results indicated in Figure 4(c) showed the appearance of a single dark-green spot with the Rf value of 0.92. The presence of photoactive compounds in the methanolic extract of flower of *P. hysterophorus* prepared at high temperature was detected by applying and resolving the extract on the TLC plate as mentioned in Materials and Methods. The dried TLC plates were exposed to UV radiation, and the spots were identified. The corresponding Rf values were 0.92 and 0.95 (Figure 4(d)). When the same plate was visualized by necked eyes, a single spot was observed with the Rf value of 0.92 representing the presence of different chemical ingredients (Figure 4(e)). The Rf values of different plant extracts as shown in Figures 1to 4 are summarized in Table 5.

### 3.6. Analysis of Free Radical Quenching Potential (DPPH) Assay of P. hysterophorus Plant Extracts Prepared at Low and High Temperatures

The plant extracts of leaves, stem, and flowers of *P. hysterophorus* prepared in hexane, ethyl acetate, methanol, and H_2_O at low temperature were tested for their free radicals quenching potential presented as described in Materials and Methods. The results presented in Table 6 indicated that the extract of *P. hysterophorus* leaves prepared in ethyl acetate at low temperature showed the maximum free radical scavenging potential followed by methanolic and aqueous extracts; the IC_50_ values being 152, 154, and 251 *μ*g/mL, respectively.

However, the leaves of *P. hysterophorus* extract prepared in hexane showed the lowest free radical quenching potential (IC_50_ 500 *μ*g/mL). When the extracts of leaves of *P. hysterophorus* were prepared in an aqueous medium and at a high temperature showed the maximum free radical scavenging potential followed by methanolic and ethyl acetate, the IC_50_ values being 65*μ*g/mL, 130 *μ*g/mL, and 248 *μ*g/mL, respectively. However, the extract prepared in hexane showed an insignificant level of free radical quenching potential (Table 6).

The extracts of *P. hysterophorus* stem prepared in ethyl acetate showed maximum inhibition potential which was almost equal to the methanolic extract prepared at low temperature, the IC_50_ values being 100 *μ*g/mL and 103 *μ*g/mL, respectively. However, no free radical scavenging activity was determined in the extracts prepared in hexane and aqueous medium. The aqueous extract of *P*. *hysterophorus* stem prepared at high temperature showed maximum inhibition potential followed by ethyl acetate extracts (IC_50_, 450 *μ*g/mL). No inhibitory potential was observed in the extracts prepared in the hexane and methanolic media (Table 6).

The extracts of *P. hysterophorus* flowers prepared at low temperature in ethyl acetate and methanolic media showed maximum free radical quenching potential, the values being 100 *μ*g/mL for both of these extracts followed by aqueous extracts (200 *μ*g/mL). However, the extract prepared in hexane at both low and high temperatures showed no quenching potential. The extract of *P. hysterophorus* flower prepared in ethyl acetate at high temperature showed maximum free radical quenching potential followed by aqueous and methanolic extracts, the values being 180 *μ*g/mL, 300 *μ*g/mL, and 350 *μ*g/mL. Free radical quenching potential was not observed in the extracts prepared in hexane (Table 6).

### 3.7. Total Antioxidant Capacity (TAC) of Extracts

**Figure 5:**
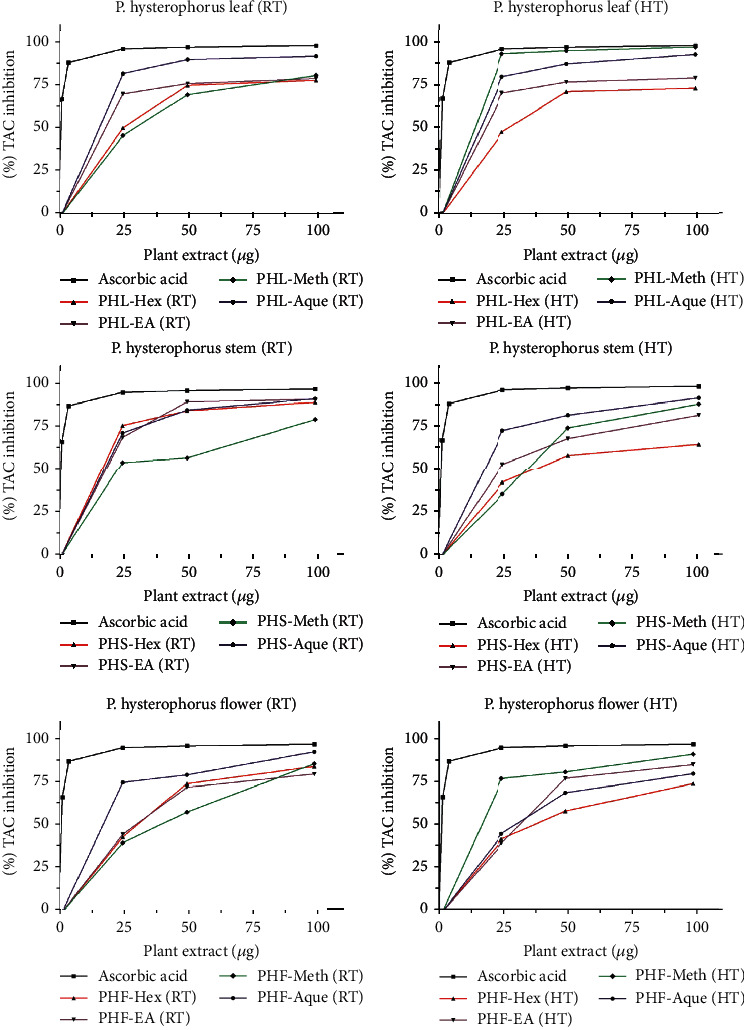
Total antioxidant capacity (TAC) of extracts of different parts of *P*. *hysterophorus* prepared in aqueous and organic solvents. PHL=P. hysterophorus leaf, PHF=P. hysterophorus flower, PHS=P. hysterophorus stem, RT=Room Temperature, HT=High temperature, Hex=Hexane, EA=Ethylacerate, Meth=Methanol, Aque=Aqueous

*P. hysterophorus* plant extracts (leaves, stem, and flowers) prepared in different solvents (hexane, ethyl acetate, methanol, and H_2_O) at low and high temperatures were tested for total antioxidant capacity (TAC). The IC_50_ value was found to be maximum (8 *μ*g/mL) for the *P. hysterophorus* leaf extract prepared at high temperature in methanolic solvent followed by extracts prepared in aqueous, ethyl acetate, and hexane with IC_50_ values 9, 10, and 29 *μ*g/mL, respectively. The lowest inhibitory concentration was recorded for *P. hysterophorus* flower extract prepared in hexane solvent at high temperature with the IC_50_ value 41 *μ*g/mL followed by *P. hysterophorus* stem extract prepared in hexane at high temperature and methanolic extracts of flower and leaves at low temperature with IC_50_ value 40, 40, and 39 *μ*g/mL, respectively. The results are shown in Figure 5 and Supplimentry Table 3.

### 3.8. The Ferric Reducing Antioxidant Power (FRAP) of Extracts

**Figure 6:**
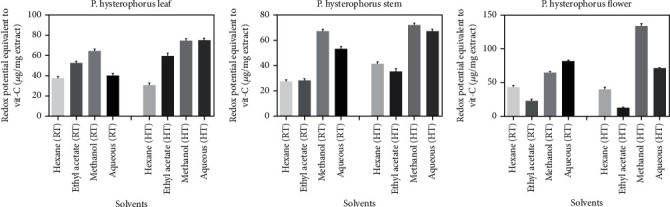
Reduction potential of the *P. hysterophorus* plant extracts prepared in different solvents at low and high temperatures.

The reduction potential of the *P. hysterophorus* extracts prepared at low and high temperatures in different solvents (hexane, ethyl acetate, methanol, and H_2_O) were tested by the ferric reducing antioxidant power (FRAP) assay. The reduction potential was shown in terms of redox potential equivalent to Vit C (*μ*g/mg extract). The reduction potential of the *P. hysterophorus* flower extract prepared in methanolic medium at high temperature showed maximum reduction potential (131.2 *μ*g/mg). The reduction potential of *P. hysterophorus* flower extract prepared in ethyl acetate medium at high temperature showed minimum reduction potential with (12 *μ*g/mg) equivalent. All other extracts showed moderate activity. The results are shown in Figure 6 and Supplimentry Table 4.

### 3.9. Evaluation of Antimicrobial Potential of Extracts of P. hysterophorus Prepared at Low and High Temperatures (Antibacterial Activity)

The antimicrobial activities of different plant extracts were determined using the procedure as described in Materials and Methods. The extracts of leaves and flowers of *P. hysterophorus* prepared in ethyl acetate, methanol, and H_2_O at low temperature were tested for their inhibitory effect against the growth of different bacterial strains such as *S. typhi*, *gonococci*, *Citrobacter*, *Flexineri*, and *S. epidermis.* The results presented in Table 7 reflected the outcomes of antimicrobial activities of different extracts against the aforesaid bacterial species. We have used both the positive (ampicillin) and negative (solvent) controls during the experiments. The antibacterial efficacy of different extracts of *P. hysterophorus* was screened by using disc diffusion method.

#### 3.9.1. Evaluation of Antimicrobial Potential of Extracts of *P. Hysterophorus* at Prepared at Low Temperature (Antibacterial Activity)

**Figure 7:**
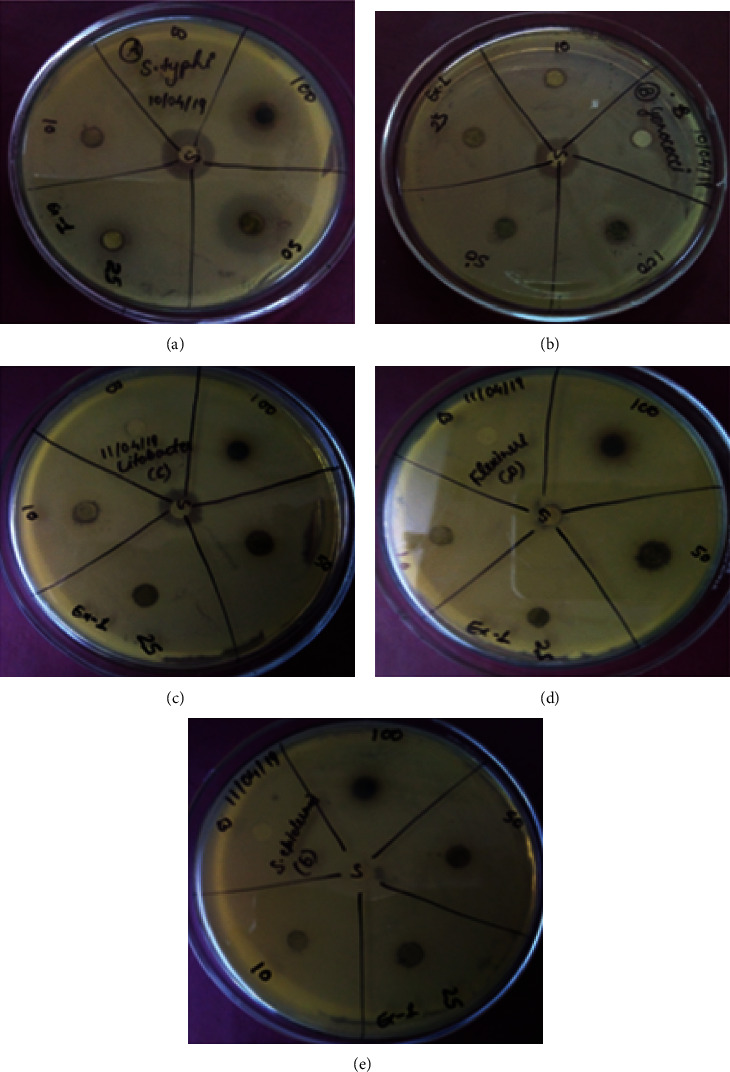
Zone of clearance by extract of *P. hysterophorus* leaves in ethyl acetate prepared at high temperature against different bacterial species: (a) *S. typhi*, (b) *gonococci*, (c) *Citrobacter*, (d) *Flexineri*, and (e). *S. epidemis.* 0, 10, 25, 50 and 100 represent the extract concentrations in µg/mL. S in the center represents ampicillin.

Among the tested bacterial species for *S. typhi*, the extract of *P. hysterophorus* leaves prepared in an aqueous solvent prepared at low temperature displayed maximum zone of inhibition **(**15 mm) at 300 *μ*g/disc concentration followed by methanolic extract of *P. hysterophorus* leaves **(**15 mm) at 500 *μ*g/disc and ethyl acetate extract of *P. hysterophorus* leaves (14 mm) at 500 *μ*g/disc. The zone of inhibition for standard (ampicillin) is 16 mm. The layout of this experiment on an LB agar plate is shown in Figure 7. The zone of clearance by different plant extracts has been presented in Table 7.

Among the tested bacterial species for *S. typhi*, the extract of *P. hysterophorus* leaves was prepared in aqueous solvent prepared at low temperature displayed maximum zone of inhibition **(**15 mm) at 300 *μ*g/disc concentration followed by methanolic extract of*P. hysterophorus* leaves **(**15 mm) at 500 *μ*g/disc and ethyl acetate extract of *P. hysterophorus* leaves (14 mm) at 500 *μ*g/disc.

The zone of inhibition for standard (ampicillin) is 16 mm. The layout of this experiment on an LB agar plate is shown in Figure 7. The zone of clearance by different plant extracts has been presented in Table 7.

#### 3.9.2. Antimicrobial Activity of Different Plant Extracts of P. hysterophorus Prepared at Low Temperature against Different Bacterial Strains

The antibacterial activities of different extracts of *P. hysterophorus* leaves and flowers prepared at room temperature in ethyl acetate, methanol, and H_2_O displayed an inhibitory effect on different bacterial species such as *S. typhi*, *gonococci*, *Citrobacter*, *Flexineri*, and *S. epidermis* growth were monitored using both the positive and negative controls as described in Materials and Methods. The results presented in Table 7 demonstrated that the extracts of *P. hysterophorus* leaves prepared in ethyl acetate at room temperature displayed a maximum zone of inhibition **(**15 mm) at 300 *μ*g/disc. Other extracts of plant leaves prepared in the methanolic and H_2_O medium at room temperature exhibited no zone of clearance against *S. typhi*. The extract of flowers of *P*. *hysterophorus* prepared in an aqueous medium displayed maximum zone of inhibition **(**8 mm) at 300 *μ*g/disc against *S*. *typhi*. However, other extracts of flowers of *P. hysterophorus* prepared in ethyl acetate and methanol showed no inhibition zone against the bacterial species. The inhibition zone for standard (ampicillin) was 17 mm at 5 mg/mL (25 *μ*g/disc). The extract of *P. hysterophorus* leaves prepared in ethyl acetate at room temperature showed maximum zone of inhibition of 13 mm at 300 *μ*g/disc. The effect was observed in the concentration-dependent manner, as lower concentration showed the lower/smaller zone of inhibition, the values being 12 mm, 11 mm, and 7 mm for 200, 100, and 50 *μ*g/disc, respectively. The rest of the extracts exhibited no zone of inhibition against the *gonococci* species. This extract displayed antimicrobial activity against *Citrobacter*; the zone of inhibition are 15 mm, 14 mm, 12 mm, 11 mm, and 6 mm for the concentrations 500, 300, 200, and 100 mg/mL, and 50 *μ*g/disc and rest of the extracts showed no zone of inhibition. The plant extract exhibited similar results against *Flexineri* sp. with zone of clearance maximum of 15 mm for the concentration 500 *μ*g/disc followed by 300 *μ*g/disc 11 mm, 10 mm, and 6 mm for the concentrations 200, 100, and 50 *μ*g/disc (Figure 7, Table 7). The zone of inhibition against *S. epidermis* species caused by this extract was also found to be 15 mm at 500 *μ*g/disc, however, at the lower concentration (Figure 7, Table 7).

Ethyl acetate medium at room temperature displayed maximum inhibition zone of 15 mm at concentrations 500 *μ*g/disc and 300 *μ*g/disc followed by 14 mm, 11 mm, and 8 mm for the concentrations 200, 100, and 50 *μ*g/disc, and the remaining extracts displayed no zone of inhibition. The extracts of flowers of *P. hysterophorus* prepared at low temperature were not effective against the tested bacterial species. However, the extract of *P. hysterophorus* flowers extract prepared in ethyl acetate at low temperature showed a zone of clearance of 11, 9, and 7 mm at concentrations of 200, 100, and 50 *μ*g/disc against gonococci (Table 7).

#### 3.9.3. Antimicrobial Activity of Different Plant Extracts of P. hysterophorus Prepared at High Temperature against Different Bacterial Strains

The extracts of different parts of *P. hysterophorus* prepared at high temperature using different solvents as mentioned above showed a similar zone of inhibition as that observed at the low temperature excepting the extract of flower of the plant prepared in ethyl acetate, which displayed a zone of inhibition 12 mm, 11 mm, 11 mm, and 11mm at 500 *μ*g/disc, 300 *μ*g/disc, and 200 *μ*g/disc concentrations, respectively, against the bacterial species *S. typhi*, *gonococci*, *Flexineri*, and *S. epidermis.* There was no zone of inhibition against *Citrobacter* for the same extract (Table 8). The extracts of *P. hysterophorus* prepared at high temperature showed a similar zone of inhibition with theextracts prepared at low temperature.

## 4. Discussion

It is reported in earlier studies that *P*. *hysterophorus* embraces so many allelochemicals/compounds which are primarily accountable for their medicinal and harmful properties. A major sesquiterpene lactone, parthenin, is mainly responsible for its allelopathic properties; allelochemicals are also present in trichomes and pollens of the plant. The presence of certain oils, histamines, terpenes, polyphenols, alkaloids, and pseudoguaianolides in *P. hysterophorus* are the main reasons for its properties associated with the health benefits [29, 30]. The extracts of *P. hysterophorus* contain polyphenols, due to which it possesses antioxidant activity. From the qualitative analysis of the plant extract, the presence of alkaloids, terpenoids, carbohydrates, and cardiac glycosides were confirmed. The Rf values obtained from thin-layer chromatography indicated the presence of more than one phytochemical compound in the *P. hysterophorus* plant extract. The *P. hysterophorus* extracts were found to show inhibitory potential against HIV-1 RT, and some bacterial strains such as *S. epidermis*, *Salmonela typhi*, *gonococci*, *Citrobacter*, and *Flexineri* species responsible for causing several diseases in humans.

UV-Vis spectrophotometric technique is proved to be a reliable and sensitive method for the detection of biomolecular compounds [31, 32]. The characteristics of molecules to absorb radiation under specific wavelengths were scanned in the entire range of 190-700 nm due to sharpness of peak and proper baseline [33]. In the present investigation, the extracts of *P. hysterophorus* prepared in different solvents of increasing polarity (hexane, ethyl acetate, methanol, and water) showed the appearance of different peaks (229, 340, 410, and 669 nm).

Unsaturated carbonyl compounds were only present/found in aqueous fractions of all the plant parts in the lower concentrations (250 *μ*g/mL) at varying wavelengths of 201, 203, 210, and 214 nm. The extracts prepared at room temperature and temperature equivalent to the boiling of the solvent (HT) indicated the presence of varying phytochemicals in different extracts as they displayed different peaks corresponding to specific wavelengths. The extracts of *P. hysterophorus* prepared at high temperature in hexane showed the presence of a common peak at wavelength 410 nm in the leaves, stem, and flowers. The extracts prepared in ethyl acetate at high temperature exhibited a common peak at 697 nm in all the parts of *P. hysterophorus* tested. In the methanolic extracts of leaves, stem, and flowers of *P. hysterophorus* commonly demonstrated the presence of a peak at 669 nm [27, 28].

Under this condition, the extracts of all theplant parts tested were exhibited absorption peaks in the UV region at the wavelengths of 330-337 nm [27, 28]. Excepting all, the extracts of leaves, stem, and flowers of *P. hysterophorus* prepared at high temperature contained flavonoids and pheophytin A as they showed absorbance of light both in the UV and visible ranges. The UV-Vis spectra of aqueous extracts of leaves, stem, and flowers of *P. hysterophorus* indicated the presence of unsaturated carbonyl compounds as well. The unsaturated compounds present in the different extracts of these plants were found to be present in aqueous extract only.

The extracts of leaves stem and flowers of *P. hysterophorus* prepared at room temperature indicated the presence of similar phytochemical as mentioned above, albeit the common peaks for these molecules in the specific part of the plant in the solvent varied. Under this condition, the extracts of leaves, stem, and flowers prepared in hexane exhibited a common peak at the wave length 670 nm representing the presence of flavonoids and pheophytin A [27, 28]. The extract of all the parts of the plants tested in ethyl acetate demonstrated a common peak at 402 nm which represents the light absorption property of flavonoids [27]. The methanolic extracts of leaves, stem, and flowers of *P . hysterophoru*s demonstrated the appearance of common peaks at around 210 nm and 342 nm representing the presence of flavonoids and pheophytin A [27, 28]. The aqueous extracts of all the parts of the *P. hysterophoru*s displayed common peaks at around 330 nm as well as 203 nm wavelengths. These results are in agreement with those reported by other workers [27, 28].

The qualitative analysis of phytochemicals isolated from different parts of *P. hysterophorus* at room temperature in all the solvents (hexane, ethyl acetate, methanol, and water) indicated the presence of terpenoids and phenols in all the preparations, whereas the flavonoids, saponins tannins, steroids, quinones, cardiac glycosides, carbohydrates, and alkaloids demonstrated varying degrees of presence in the same. Among these phytochemicals, flavonoid, saponin, and tannins were observed to be present in a very small amount. Cardiac glycosides could not be detected in the aqueous and methanolic extracts whereas the hexane and ethyl acetate showed the presence of a substantial amount of cardiac glycoside in the leaves, stem, and flowers (present investigation, Table 1). When the qualitative analysis of the phytochemicals present in the extracts of the different parts of the *P. physterophorus* prepared at high temperature was carried out, the flavonoids, saponins, and tannins were either not detected or were present in very small amounts in the preparations using hexane, ethyl acetate, and methanol; however, the aqueous extracts showed the presence of these molecules in leaves and stem (Table 4). The similar findings have been reported by other workers in the same plant [34–36]. They have also shown the presence of these phytochemicals in the root of *P. hysterophosus*.

We have tested the antiviral properties of the extracts of *P. hysterophorus*, and it was observed that the extract of leaves of the plant prepared in aqueous medium at room temperature exhibited very strong anti-HIV potential with IC_50_ value being 0.081 mg/mL followed by the ethyl acetate extract of flower at the high temperature (0.31 mg/mL) in comparison to other extracts of the plant [37]. In the present study, the order of antiviral activity of other extracts from leaves of plant was as follows: ethyl acetate extract prepared at room temperature (IC_50_-0.34 mg/mL)>extracts prepared at high temperature in aqueous (IC_50_-0.40 mg/mL)>and hexane (IC_50_-0.44 mg/mL). However, the extracts of the flowers prepared at high temperature indicated the order of their activities against HIV-1 RT as follows: ethyl acetate extract (IC_50_-0.40 mg/mL) and methanolic extract (IC_50_-0.47 mg/mL). The comparison of the above data suggested that the anti-HIV-1 activity of the extracts of leaves prepared in the aqueous medium at room temperature was most active among all the extracts [38].

TLC is a technique for the separation and identification of natural substances and is utilized to resolve the low molecular weight compounds according to their polarity and partial distribution in mobile phase. The application of TLC is a common practice to isolate phytochemicals from different plant extracts. In the above section, the extracts of flowers prepared in ethyl acetate and methanol at room and high temperature, respectively, indicated significant inhibition of HIV-1 RT inhibition activity; also, the extracts of leaves of *P. hysterophorus* prepared in water and ethyl acetate at room temperature were also active against HIV-1 RT activity. We have therefore analyzed the phytochemicals present in these extracts using TLC. The application of specific reagents in the TLC plates indicated the presence of flavonoids as well as primary and secondary amines (Dragendorff's reagent): antioxidants, steroids, prostaglandins, carbohydrates, phenols, saponins, mayotoxins, essential oils, and terpenes (anisaldhyde–sulphuric acid reagent); flavonoids (Mayer's reagent); compounds absorbing visible and UV radiation (UV *trans*-illuminator); and visible light.

In the present study, it was examined that the lower Rf value of flavonoids was due to its polarity which was highest among all, in comparison with amines which were less polar than flavonoids and travel a little higher than them. Among all of them, some compounds such as carbohydrates, glycosides, phenols, and many antioxidants, being polar, showed lower Rf values than those which were polar in nature such as steroids, prostaglandins, essential oils, and terpenes. Some compounds with intermediate Rf values such as saponins and myotoxin had both polar and nonpolar natures. Pande et al. have shown the Rf values for phenol, flavonoid, and alkaloids from leaves of *P*. *hysterophorus* were 0.83, 0.45, and 0.9, respectively. These researchers have expressed that Rf values ranging from 0.3 to 0.9 correspond to the presence of terpenes or terpenoids [39].

Like many other medicinally important plants, the extracts of *P. hysterophorus* leaves showed antimicrobial activity; the extract of leaves prepared in ethyl acetate at room temperature exhibited a maximum zone of inhibition **(**15 mm) at 300 *μ*g/disc. Other extracts of plant leaves prepared in the methanolic and aqueous medium at room temperature exhibited no zone of clearance against *S. typhi* (present investigation). In contrast, the extract of flower of the plant in aqueous medium at room temperature exhibited maximum antimicrobial activity, zone of inhibition being **(**8 mm) at 300 *μ*g/disc against *S. typhi.* Other extracts of flowers of this plant did not show antimicrobial activity. Similar findings have been reported by other workers [38, 40]. The essential oil extracted from Asian ginseng leaves, which contained a complex mixture of aliphatic (69.0%), terpenoid (21.5%), and aromatic compounds (2.4%), showed antibacterial activity against the Gram-positive bacteria *S. aureus* and *B. subtilis* and the Gram-negative bacterium, *E. coli*, with the inhibition zone of 13.7, 14.0, and 15.0 mm, respectively [41, 42]. The extracts of *P. hysterophorus* prepared in different solvents: hexane, ethyl acetate, methanol, and water, at their respective boiling points, were tested against bacterial species such as Gram-negative bacteria (*S. typhi*, *N. gonorrhoeae* or *gonococci*, *Citrobacter*, and *S. flexneri*) and Gram-positive bacterium (*S. epidermidis*).

*P. hysterophorus* leaf extract prepared at high temperatures showed maximum free radical quenching potential (using DPPH method) withIC_50_ value of 65 *μ*g/mL, whereas the leaves of *P. hysterophorus* extract prepared in hexane possess the lowest free radical quenching potential with IC_50_ value of 500 *μ*g/mL. According to Ahmad et al. (2018), the root extract of *P. hysterophorus* possesses maxiumum free radical scavenging activity which was followed by extracts of receptacle, leaf, and stem. The possible free radical scavenging activity is due to the presence of a range of terpenes, sterols, and fatty acids in the extracts of *P. hysterophorus*. Among all these secondary metabolites terpenes and terpenoids are major groups contributing to the free radical scavenging activity [43]. Methanolic extracts of *P. hysterophorus* showed significant antioxidant activity as compared to the ascorbic acid. This might be due to the presence of bioactive elements and phenolic constituents present in the extract [44].

Among all the tested extracts for total antioxidant capacity (TAC), the *P. hysterophorus* leaf extract prepared at high temperature in methanolic solvents shown maximum antioxidant capacity. In a study done by Ahmad et al., total antioxidant capacity (TAC) of *P. hysterophorus* increased with increased duration of stress in leaves; the leaves exposed to high light (HL) showed maximum TAC as compared to leaves exposed to medium light (ML) [11]. The reduction potential of the *P. hysterophorus* extract was maximum in flower extract prepared in the methanolic medium at high temperature. The FRAP assay represented direct correlation between high reducing power and high content of phytoconstituents in receptacle and root extract [43].

Rai and Lall have reported that the copper nanoparticles synthesized with *P. hysterophorus* leaves have more free radical scavenging activity (67%) in comparison with methanolic extracts of leaves prepared at room temperature (28.82%) and standard ascorbic acid (44.85%) [45]. They have indicated that copper nanoparticles can be an effective antioxidant agent. It is predicted that higher antioxidant activity of nanoparticles might be due to encapsulation of bioactive molecules on the surface of CuNPs through the electrostatic attraction between negatively charged bioactive compounds (COO-, O-) and neutral or positively charged nanoparticles [45, 46].

In the present study, spectrometric and qualitative analysis showed that terpenoides and quinones are also present in major quantities in the extracts of different parts of the *P. hysterophorus*. Terpenoids are the precursor for the synthesis of different terpenes and sesquiterpenes. Parthenin which is a characteristic compound of *P. hysterophorus* is a sesquiterpene. Quinones also have the structure-wise similarity with terpenes. In our study, it was observed that the extracts containing a higher amount of terpenoids and quinones showed more inhibitory activity against HIV-1 RT. One of the reasons could be the interaction of these compounds with HIV-1 RT protein responsible for the inhibition of enzyme function.

## 5. Conclusion

The results from this study suggested that the crude extracts contained sufficient amount of phytochemicals/allelochemicals, which were responsible for their medicinal and toxicological properties. The qualitative analyses of the plant extracts demonstrated the presence of alkaloids, flavanoids, terpenoids, carbohydrates, quinines, and cardiac glycosides. The crude extracts of the plant in different solvents displayed sufficient antioxidant activity. The Rf values of the extracts determined by TLC indicated the appearance of more than one Rf value for any single extract, which represented the presence of more than one phytochemical compound in the preparation. The major phytochemicals present in the extracts were responsible for their active contribution towards inhibition of growth of some bacterial species such as *S. typhi*, *gonococci*, *Citrobacter*, *Flexiner*, and *S. epidermis.* The results demonstrated that the extracts of *P. hysterophorus* have phytochemicals with potential inhibitory activity against microbes and HIV-1 RT. As compared to a previous study of Kumar et al. (2014), it was observed that our aqueous leaf extract showed an IC_50_ value at 81 *μ*g, which on their case was an observation of 38% inhibition at 6 *μ*g/mL of aqueous leaf extract; in another study done by Ishizuka et al. (2020), 50% inhibition was observed at 60.4*μ*g in the aqueous leaf extract.

## Figures and Tables

**Table 1:** Qualitative phytochemical analysis of *P. hysterophorus* leaf, stem, and flower at low temperature.

Plant name	Solvent	Phytochemicals	Leaf	Stem	Flower
*P. hysterophorus*	Hexane	Flavonoids	ND	ND	ND
		Saponins	ND	ND	ND
		Tannins	ND	ND	ND
		Terpenoids	+ + + + +	+ + + +	+ + + +
		Steroids	+ +	+	+ +
		Phenols	+ + +	+ + +	+
		Quinones	+ + +	+ +	+ +
		Cardiac glycosides	+ + +	+ +	+ +
		Carbohydrates	ND	+ + +	+ + +
		Alkaloids	+	ND	ND
	Ethyl acetate	Flavonoids	+ +	+	ND
		Saponins	ND	ND	ND
		Tannins	ND	ND	ND
		Terpenoids	+ + + +	+ + + +	+ + + +
		Steroids	+ + +	+	+ +
		Phenols	+ + +	+ + +	+
		Quinones	+ + +	+ + + +	+ +
		Cardiac glycosides	+ + +	+ + +	+ + + +
		Carbohydrates	+ +	+ +	+ + +
		Alkaloids	ND	+ + +	ND
	Methanol	Flavonoids	ND	ND	ND
		Saponins	+	—	+
		Tannins	+ +	+ +	ND
		Terpenoids	+ + +	++	+ + + +
		Steroids	+	ND	+ +
		Phenols	+ + +	+	+ +
		Quinones	ND	ND	+ +
		Cardiac glycosides	+ +	ND	ND
		Carbohydrates	+ + +	+ + +	+ + + +
		Alkaloids	+ + + +	ND	+ + + +
	Aqueous	Flavonoids	+	+	ND
		Saponins	+ +	ND	ND
		Tannins	ND	ND	+ + +
		Terpenoids	+ + + + +	+ +	+ + + +
		Steroids	+ +	+	+ +
		Phenols	+	+	+ +
		Quinones	+ + +	ND	+
		Cardiac glycosides	ND	ND	ND
		Carbohydrates	+ +	ND	+
		Alkaloids	+ + + +	+ + +	+ + + + +

^#^The sign (+) shows the presence of phytochemicals in the extract, and the increase in the sign (+) shows the presence of more amount of phytochemicals. ND represents the compounds not detected.

**Table 2:** Qualitative phytochemical analysis of *P. hysterophorus* leaf stem and flower extracts prepared at high temperature.

Plant name	Solvent	Phytochemicals	Leaf	Stem	Flower
*P. hysterophorus*	Hexane	Flavonoids	ND	ND	ND
		Saponins	ND	ND	ND
		Tannins	ND	ND	ND
		Terpenoids	+	+	+ + +
		Steroids	+ +	+ +	++
		Phenols	+ +	+ +	+ +
		Quinones	+ + + +	+ + + +	+ +
		Cardiac glycosides	+ +	+ + +	+ +
		Carbohydrates	ND	+ + +	+ + + +
		Alkaloids	ND	ND	+
	Ethyl acetate	Flavonoids	ND	ND	+ +
		Saponins	ND	ND	ND
		Tannins	ND	ND	ND
		Terpenoids	+ + + +	+ + + +	+ + +
		Steroids	+ + +	+ + +	+ +
		Phenols	+ + +	+ + +	+ + + +
		Quinones	+ + +	+ + +	+ + +
		Cardiac glycosides	+ +	+ + + +	+ + + +
		Carbohydrates	+ + +	+ +	+ + + +
		Alkaloids	ND	+	+
	Methanol	Flavonoids	ND	ND	ND
		Saponins	+	+	ND
		Tannins	ND	ND	ND
		Terpenoids	+ + +	+ + +	+ + +
		Steroids	ND	ND	++
		Phenols	+ +	+ +	+
		Quinones	ND	ND	+ + +
		Cardiac glycosides	+ + +	ND	+ +
		Carbohydrates	+ + +	+ + +	+
		Alkaloids	+ + +	+ +	+ + +
	Aqueous	Flavonoids	+ + +	+ + +	ND
		Saponins	+ +	+ +	+
		Tannins	+ + +	+ + +	+ + +
		Terpenoids	+	+	+ +
		Steroids	ND	ND	++
		Phenols	+ + +	+ + +	+ +
		Quinones	+ +	+ +	++
		Cardiac glycosides	ND	ND	ND
		Carbohydrates	+ +	+ +	ND
		Alkaloids	+ + +	+ +	+ + +

^#^The sign (+) shows the presence of phytochemicals in the extract, and the increase in the sign (+) shows presence of more amount of phytochemicals. ND represents the compounds not detected.

**Table 3:** Anti-HIV-1 RT activities of *P. hysterophorus* leaves extracts prepared at high temperature and low (room temperature**)** temperatures.

Plant name and part	Solvents	Extract (mg/mL)	Inhibition of activity (%)	IC_50_ (mg/mL)
*P. hysterophorus* leaves	Hexane (HT)	0.083	11	0.44
		0.166	24	
		0.332	34	
		0.664	70	
	Ethyl acetate (HT)	0.083	8	0.55
		0.166	17	
		0.332	29	
		0.664	60	
	Methanol (HT)	0.083	4	ND
		0.166	7	
		0.332	15	
		0.664	25	
	Aqueous (HT)	0.083	11	0.40
		0.166	25	
		0.332	47	
		0.664	83	
	Hexane (RT)	0.083	9	0.65
		0.166	19	
		0.332	26	
		0.664	45	
	Ethyl acetate (RT)	0.083	14	0.34
		0.166	31	
		0.332	52	
		0.664	93	
	Methanol (RT)	0.083	14	ND
		0.166	14	
		0.332	30	
		0.664	67	
	Aqueous (RT)	0.083	51	0.081
		0.166	87	
		0.332	100	

^#^ND: not determined; RT: room temperature; HT: high temperature.

**Table 4:** Anti-HIV-1 RT activities of *P. hysterophorus* flowers extracts prepared at high temperature and low temperatures.

Plant name and part	Solvent	Extract concentration (*μ*g)	Activity lost (% inhibition)	IC_50_ (mg/mL)
*P. hysterophorus* flowers	Ethyl acetate (HT)	0.083	15	0.31
		0.166	35	
		0.332	56	
		0.664	100	
	Methanol (HT)	0.083	8	0.47
		0.166	17	
		0.332	32	
		0.664	67	
	Aqueous (HT)	0.083	0	ND
		0.166	0	
		0.332	9	
		0.664	15	
	Ethyl acetate (RT)	0.083	8	0.40
		0.166	18	
		0.332	44	
		0.664	78	
	Methanol (RT)	0.083	5	ND
		0.166	10	
		0.332	25	
		0.664	47	
	Aqueous (RT)	0.083	3	ND
		0.166	18	
		0.332	16	
		0.664	25	

^#^ND: not determined; RT: room temperature; HT: high temperature.

**Table 5:** Rf values of spots obtained using various physical and chemical methods for different parts of plant extracts as shown in Figures 1–4.

S. no.	Plant name	Rf values of phytochemicals determined by Dragendorff reagent	Rf values of phytochemicals determined by *λ* anisaldehyde- sulphuric acid reagent	Rf values of phytochemicals determined by Mayer's reagent	Rf values of phytochemicals determined by UV light	Rf values of phytochemicals determined in visible light
1	*P*. *hysterophorus* flower RT-Ea	0.66, 0.73, 0.93	0.66, 0.93, 0.96	0.66, 0.93	0.66, 0.93	0.66, 0.93
2	*P*. *hysterophorus* leaf RT-Aqu	0.75, 0.92	0.75, 0.85, 0.92	0.92	0.85, 0.92	0.92
3	*P*. *hysterophorus* leaf RT-Ea	0.86, 0.93	0.45, 0.86, 0.93	0.53, 0.86	0.53, 0.86	0.53, 0.86
4	*P. hysterophorus* flower HT-meth	0.80, 0.92	0.95	0.92	0.92, 0.97	0.92, 0.98

^#^The Rf values of different phytochemicals prepared in varying solvents have been calculated through TLC. RT=Room temperature, Ea=Ethylacetate, Aqu=Aqueous, Meth=methanol.

**Table 6:** Free radical quenching potential of different extracts of *P*. *hysterophorus* prepared at low and high temperatures.

*P. hysterophorus*	Solvent	IC_50_ (*μ*g/mL) of extracts	
		Low temperature	High temperature
Leaf	Hexane	500	ND
	Ethyl acetate	152	248
	Methanol	154	130
	Aqueous	251	65
Stem	Hexane	ND	ND
	Ethyl acetate	100	450
	Methanol	103	ND
	Aqueous	ND	275
Flower	Hexane	ND	ND
	Ethyl acetate	100	180
	Methanol	100	350
	Aqueous	200	300

^#^IC_50_ is the concentration of plant extract to neutralize the free radicals by 50%. ND: not determined.

**Table 7:** Antimicrobial activities of different extracts of leaves and flowers of *P. hysterophorus* prepared at low temperature.

Part of the plant	Solvents	Bacterial strain	Zone of inhibition (mm)						
			Concentration of extracts (mg/mL)						
			0	50	100	200	300	500	S
*P. hysterophorus* leaf	Ethyl acetate	*Salmonela typhi* (A)	0	—	8	—	15	—	16
	Methanol		0	—	—	—	—	—	15
	Aqueous		0		—	—	—	—	14
*P. hysterophorus* flower	Ethyl acetate		0		—	—	—	—	15
	Methanol		0		—	—	—	—	14
	Aqueous		0	—	6	—	8	—	17
*P. hysterophorus* leaf	Ethyl acetate	*Gonococci* (B)	0	7	11	12	13	—	14
	Methanol		0	—	—	—	—	—	12
	Aqueous		0		—	—	—	—	15
*P. hysterophorus* flower	Ethyl acetate		0	—	—	—	—	—	14
	Methanol		0	—	—	—	—	—	15
	Aqueous		0	—	—	—	—	—	14
*P. hysterophorus* leaf	Ethyl acetate	*Citrobacter* (C)	0	6	11	12	14	15	15
	Methanol		0	—	—	—	—	—	14
	Aqueous			—	—	—	—	—	14
*P. hysterophorus* flower	Ethyl acetate		0	—	—	—	—	—	14
	Methanol		0	—	—	—	—	—	15
	Aqueous		0	—	—	—	—	—	12
*P. hysterophorus* leaf	Ethyl acetate	*Flexineri* (D)	0	6	10	11	15	15	16
	Methanol		0	—	—	—	—	—	14
	Aqueous		0	—	—	—	—	—	14
*P. hysterophorus* flower	Ethyl acetate		0	—	—	—	—	—	13
	Methanol		0	—	—	—	—	—	14
	Aqueous		0	—	—	—	—	—	14
*P. hysterophorus* leaf	Ethyl acetate	*S. epidermis* (E)	0	8	11	14	15	15	15
	Methanol		0	—	—	—	—	—	13
	Aqueous			—	—	—	—	—	14
*P. hysterophorus* flower	Ethyl acetate		0	—	—	—	—	—	15
	Methanol		0	—	—	—	—	—	14
	Aqueous		0	—	—	—	—	—	14

^#^S: standard (ampicillin).

**Table 8:** Antimicrobial activity of different plant extracts of *P*. *hysterophorus* prepared at high temperature against different bacterial strains.

Plant name and part	Solvents	Bacterial strain	Zone of inhibition (mm)						
			Concentration of extracts (mg/mL)						
			0	50	100	200	300	500	S
*P. hysterophorus leaf*	Ethyl acetate	*Salmonela typhi* (A)	0	7	9	—	—	14	15
	Methanol		0	6	7	—	—	15	16
	Aqueous		0	6	10	13	15	—	15
*P. hysterophorus* flower	Ethyl acetate		0	6	8	10	10	12	14
	Methanol		0	—	—	—	—	—	13
	Aqueous		0	—	—	—	—		14
*P. hysterophorus leaf*	Ethyl acetate	*Gonococci* (B)	—	6	7	—	—	—	14
	Methanol		—	—	—	—	—	-.	12
	Aqueous		0	—	—	—	—	—	12
*P. hysterophorus* flower	Ethyl acetate		0	6	7	—	11	—	13
	Methanol		0	—	—	—	—	—	14
	Aqueous		—	—	—	—	—	—	13
*P. hysterophorus* leaf	Ethyl acetate	*Citrobacter* (C)	—	6	6	—	—		14
	Methanol		—	—	—	—	—	—	14
	Aqueous		0	—	—	—	—	—	14
*P. hysterophorus* flower	Ethyl acetate		0	—	—	—	—	—	12
	Methanol		0	—	—	—	—	—	14
	Aqueous		—	—	—	—	—	—	14
*P. hysterophorus* leaf	Ethyl acetate	*Flexineri* (D)	0	7	8	—	—	—	13
	Methanol		—	—	—	—	—	—	14
	Aqueous		—	—	—	—	—	—	14
*P. hysterophorus* flower	Ethyl acetate		—	7	7	11	11	—	13
	Methanol		—	—	—	—	—	—	13
	Aqueous		—	—	—	—	—	—	15
*P. hysterophorus* leaf	Ethyl acetate	*S. epidermis* (E)	0	—	7	9	—	—	14
	Methanol		0	—	8	11	13	13	15
	Aqueous		—	—	—	—	—	—	16
*P. hysterophorus* flower	Ethyl acetate		—	—	7	11	11	—	14
	Methanol		—	—	—	—	—	—	15
	Aqueous		—	—	—	—	—	—	14

S: standard (ampicillin).

## Data Availability

The main data used to support the findings of this study are included in the article. A summary of the analysis of different absorption peaks generated due to absorption of light in the UV-visible regions by different phytochemicals present in the extracts of leaves, stem, and flowers of P. hysterophorus in varying solvents at high temperature (equivalent to their boiling points).

## Supplementary Materials

Supplementary Materials Supplementary Table 1: a summary of analysis of different absorption peaks generated due to absorption of light in the UV-visible regions by different phytochemicals present in the extracts of leaves, stem, and flowers of *P. hysterophorus* in varying solvents at high temperature (equivalent to their boiling points). SupplementaryTable2: A summary of analysis of different absorption peaks generated due to absorption of light in the UV-visible regions by different phytochemicals present in the extracts of leaves, stem, and flowers of *P. hysterophorus* in varying solvents at low temperature (equivalent to room temperature, i.e., 24 ± 2°C). Supplementary Table 3: IC_50_ values for total antioxidant capacity of *P. hysterophorus* extracts prepared in high and low temperatures in different solvents. Supplementary Table 4: reduction potential of the *P. hysterophorus* plant extracts prepared at high and low temperatures. Supplementary Figure 1: spectrophotometric analysis of the extracts of leaves, stem, and flowers of *P. hysterophorus* prepared in different solvents at high temperature using Soxhlet apparatus showing their varying absorption abilities of light in the UV-visible range. Solvents used were as follows: A: hexane, B: ethyl acetate, C: methanol, and D: aqueous. Supplementary Figure 2: spectrophotometric analysis of the extracts of leaves, stem, and flowers of *P. hysterophorus* prepared in different solvents at low temperature using Soxhlet apparatus showing their varying absorption abilities of light in the UV-visible range. Solvents used were as follows: A: hexane, B: ethyl acetate, C: methanol, and D: aqueous.
