# Supplementary material for: Biochemical Characterization of Different Chemical Components of Parthenium hysterophorus and Their Therapeutic Potential against HIV-1 RT and Microbial Growth
Source: Biomed Res Int. 2022 Apr 28;2022:3892352. doi: 10.1155/2022/3892352 (PMC9071890; doi:10.1155/2022/3892352)
Supplement: Supplementary Materials — Supplementary Table 1: a summary of analysis of different absorption peaks generated due to absorption of light in the UV-visible regions by different phytochemicals present in the extracts of leaves, stem, and flowers of P. hysterophorus in varying solvents at high temperature (equivalent to their boiling points). SupplementaryTable2: A summary of analysis of different absorption peaks generated due to absorption of light in the UV-visible regions by different phytochemicals present in the extracts of leaves, stem, and flowers of P. hysterophorus in varying solvents at low temperature (equivalent to room temperature, i.e., 24 ± 2°C). Supplementary Table 3: IC50 values for total antioxidant capacity of P. hysterophorus extracts prepared in high and low temperatures in different solvents. Supplementary Table 4: reduction potential of the P. hysterophorus plant extracts prepared at high and low temperatures. Supplementary Figure 1: spectrophotometric analysis of the extracts of leaves, stem, and flowers of P. hysterophorus prepared in different solvents at high temperature using Soxhlet apparatus showing their varying absorption abilities of light in the UV-visible range. Solvents used were as follows: A: hexane, B: ethyl acetate, C: methanol, and D: aqueous. Supplementary Figure 2: spectrophotometric analysis of the extracts of leaves, stem, and flowers of P. hysterophorus prepared in different solvents at low temperature using Soxhlet apparatus showing their varying absorption abilities of light in the UV-visible range. Solvents used were as follows: A: hexane, B: ethyl acetate, C: methanol, and D: aqueous. [file 3892352.f1.pdf]

Supplementary Materials

Biochemical Characterization of different Chemical Components of *Parthenium hysterophorus* and their Therapeutic Potential gainst HIV-1 RT and Microbial growth

Jyotsna Jaiswal[1], Pawan Kumar Doharey[1], Reetika Singh[1], Priyanka Tiwari[1], Nitika Singh[1], Abhishek Kumar[1], Vivek KumarGupta[1], Arif Jamal Siddiqui[2], Bechan Sharma[1*]

[1]Department of Biochemistry, Faculty of Science, University of Allahabad, Prayagraj-211002, India.

[2]Department of Biology, College of Science, University of Hail, Hail, P.O. Box 2440, Saudi Arabia.

[*]Corresponding author: Email: sharmabi@yahoo.com; Contact: +91-9415715639
Co-corresponding author: arifjamal13@gmail.com

Supplementary Tables

SUPPLEMENTARY TABLE 1: A summary of analysis of different absorption peaks generated due to absorption of light in the UV-Visible regions by different phytochemicals present in the extracts of leaves, stem, and flowers of *P. hysterophorus* in varying solvents at high temperature (equivalent to their boiling points)

Plant name	Part of plant	Solvents	Extract (mg/ml)	No. of peaks	Wavelength	Probable phytochemical	Reference
Parthenium hysterophorus	Leaf	Hexane	5 mg/ml	4	229, 340, 410, 669	Terpenoids, Flavonoids, Pheophytin A	[27, 28]
			250µg/ml	4	229, 340, 410, 669	Terpenoids, Flavonoids, Pheophytin A	[27, 28]
		Ethyl acetate	5 mg/ml	3	415, 697	Flavonoids, Pheophytin A	[27, 28]
			250µg/ml	3	407, 697	Flavonoids, Pheophytin A	[27, 28]
		Methanol	5 mg/ml		345, 389, 669	Flavonoids, Pheophytin A	[27, 28]
			250µg/ml		331, 408, 664	Flavonoids, Pheophytin A	[27, 28]
		Aqueous	5 mg/ml		231, 337, 360	Terpenoids, Flavonoids, Pheophytin A	[27, 28]
			250µg/ml		201	Unsaturated carbonyl compounds	Not any
	Stem	Hexane	5 mg/ml	3	351, 410, 669	Flavonoids, Pheophytin A	[27, 28]

			250µg/ml	3	226, 410 ,669	Terpenoids, Flavonoids, Pheophytin A	[27, 28]
		Ethyl acetate	5 mg/ml		402, 648, 655	Flavonoids,	[27, 28]
			250µg/ml		484, 576, 697	Flavonoids, Pheophytin A	[27, 28]
		Methanol	5 mg/ml		317, 379, 669	Flavonoids, Pheophytin A	[27, 28]
			250µg/ml		496	Flavonoids,	[27]
		Aqueous	5 mg/ml		335, 350	Flavonoids,	[27]
			250µg/ml		210, 214	Unsaturated carbonyl compounds	Not any
	Flower	Hexane	5 mg/ml	3	365, 385, 670	Flavonoids, Pheophytin A	[27, 28]
			250µg/ml	3	226, 411, 669	Terpenoids, Flavonoids, Pheophytin A	[27, 28]
		Ethyl acetate	5 mg/ml	3	367, 427, 664	Flavonoids, Pheophytin A	[27, 28]
			250µg/ml	3	484, 576, 697	Flavonoids, Pheophytin A	[27, 28]
		Methanol	5 mg/ml	3	207, 384, 669	Flavonoids, Pheophytin A	[27, 28]
			250µg/ml	1	207	Unsaturated carbonyl compounds	Not any
		Aqueous	5 mg/ml	2	330, 352	Flavonoids	[27]
			250µg/ml	1	203	Unsaturated carbonyl compounds	Not any

The phytochemicals shown in the Table comprise a family of compounds which show absorbance of light at different wavelengths in UV-visible regions.

The results of spectrophotometric analysis of different phytochemicals present in different extracts of leaves, stem and, flowers prepared at room temperature (24 ± 2^{0}C) are presented in Supplementary Table 2.

SUPPLEMENTARY TABLE 2: A summary of analysis of different absorption peaks generated due to absorption of light in the UV-Visible regions by different phytochemicals present in the extracts of leaves, stem and flowers of *P. hysterophorus* in varying solvents at low temperature (equivalent to room temperature i.e., 24±2^{0}C)

Plant name	Part of plant	Solvents	Extract (mg/ml)	No. of peaks	Wavelength	Probable phytochemical	Reference
Parthenium hysterophorus	Leaf	Hexane	5 mg/ml	5	380, 410, 533, 669	Flavonoids, Pheophytin A	[27, 28]
			250µg/ml	4	399,670	Flavonoids, Pheophytin A	[27, 28]
		Ethyl acetate	5 mg/ml	3	415,655	Flavonoids, Pheophytin A	[27, 28]
			250µg/ml	3	402, 576, 654	Flavonoids	[27]
		Methanol	5 mg/ml	3	340, 399, 667	Flavonoids, Pheophytin A	[27, 28]
			250µg/ml	2	218, 331	Flavonoids, Pheophytin A	[27, 28]
		Aqueous	5 mg/ml	2	194, 329, 374	Flavonoids	[27]
			250µg/ml		201	Unsaturated carbonyl compounds	Not any
	Stem	Hexane	5 mg/ml	3	399, 670	Flavonoids, Pheophytin A	[27, 28]
			250µg/ml	3	410, 669	Flavonoids, Pheophytin A	[27,28]
		Ethyl acetate	5 mg/ml		388, 653	Flavonoids	[27]
			250µg/ml		576, 697	Flavonoids, Pheophytin A	[27, 28]
		Methanol	5 mg/ml		210, 342	Flavonoids, Pheophytin A	[27, 28]
			250µg/ml		208	Flavonoids	[27]
		Aqueous	5 mg/ml		225, 331	Flavonoids	[27]
			250µg/ml		209	Unsaturated carbonyl compounds	Not any
	Flow	Hexane	5 mg/ml	3	384, 670	Flavonoids, Pheophytin A	[27, 28]
			250µg/ml	3	307, 669	Flavonoids,	[27, 28]

er					Pheophytin A	
	Ethyl acetate	5 mg/ml	3	379, 402, 667	Flavonoids, Pheophytin A	[27, 28]
		250µg/ml	2	402, 669	Flavonoids, Pheophytin A	[27, 28]
	Methanol	5 mg/ml	3	346, 383, 669	Flavonoids, Pheophytin A	[27, 28]
		250µg/ml	1	213	Unsaturated carbonyl compounds	[27, 28]
	Aqueous	5 mg/ml	2	330, 352	Flavonoids	[27, 28]
		250µg/ml	1	203	Unsaturated carbonyl compounds	Not any

The phytochemicals shown in the Table comprise a family of compounds which show absorbance of light at different wavelengths in UV-visible regions.

SUPPLEMENTARY TABLE 3: IC_{50} values for total antioxidant capacity of *P. hysterophorus* extracts prepared in high and low temperatures in different solvents

P. hysterophorus	Solvent	IC_{50} (µg/ml) of extracts	
		Low temperature	High Temperature
Leaf	Hexane	27	29
	Ethyl acetate	15	10
	Methanol	39	8
	Aqueous	14	9
Stem	Hexane	13	40
	Ethyl acetate	13	25
	Methanol	24	36
	Aqueous	14	15
Flower	Hexane	28	41
	Ethyl acetate	28	30
	Methanol	40	12
	Aqueous	13	30

SUPPLEMENTARY TABLE 4: Reduction potential of the *P. hysterophorus* plant extracts prepared at high and low temperatures

S. No.	*P. hysterophorus*	Solvent	Redox potential Equivalent to Vit-C (µg / mg Extract)
1	Leaf (Low temperature, RT)	Hexane	36
2		Ethyl acetate	52
3		Methanol	64
4		Aqueous	40
5	Leaf (High temperature, HT)	Hexane	30.4
6		Ethyl acetate	60
7		Methanol	75
8		Aqueous	75
9	Stem (Low temperature, RT)	Hexane	27
10		Ethyl acetate	27
11		Methanol	67.2
12		Aqueous	52
13	Stem (High temperature, HT)	Hexane	41
14		Ethyl acetate	36
15		Methanol	73.6
16		Aqueous	67.2
17	Flower (Low temperature, RT)	Hexane	43.2
18		Ethyl acetate	22.4
19		Methanol	64
20		Aqueous	83
21	Flower (High temperature, HT)	Hexane	38
22		Ethyl acetate	12
23		Methanol	131.2
24		Aqueous	70

Supplementary Figures

Supplementary Figure 1:

Leaf

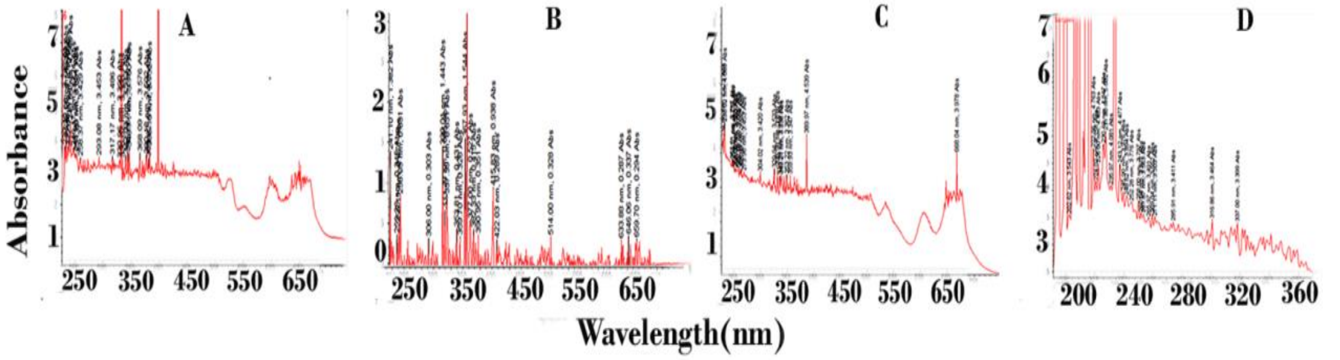

Stem

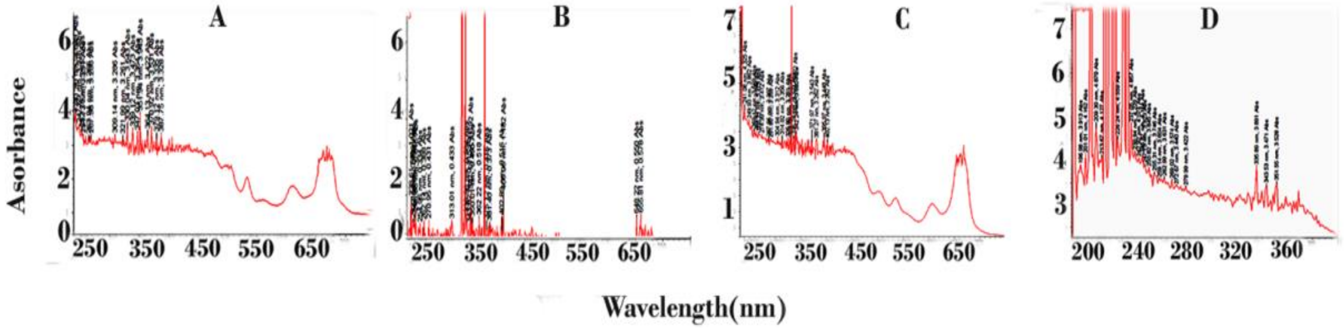

Flower

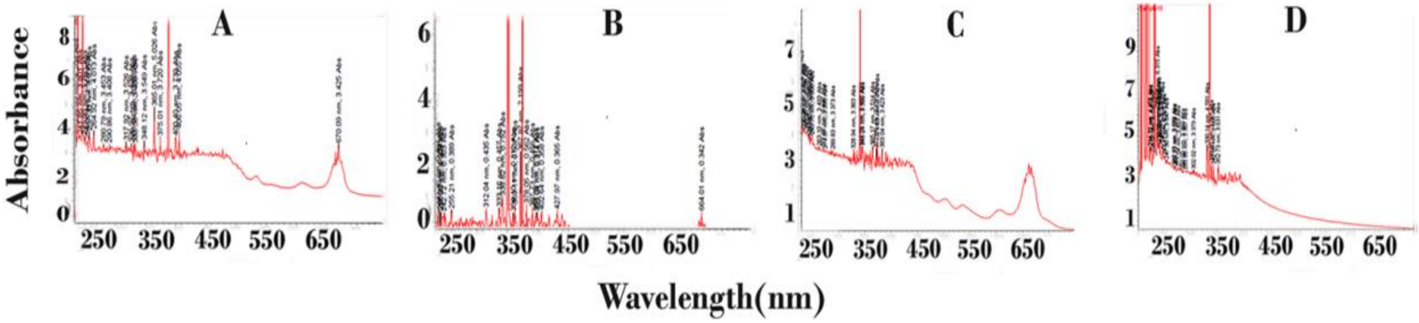

Supplementary Figure 1: Spectrophotometric analysis of the extracts of leaves, stem and flowers of *P. hysterophorus* prepared in different solvents at high temperature using Soxhlet apparatus showing their varying absorption abilities of light in the UV-Visible range. Solvents used were A=Hexane, B= Ethyl acetate, C=Methanol, and D=Aqueous

Supplementary Figure 2:

Leaf

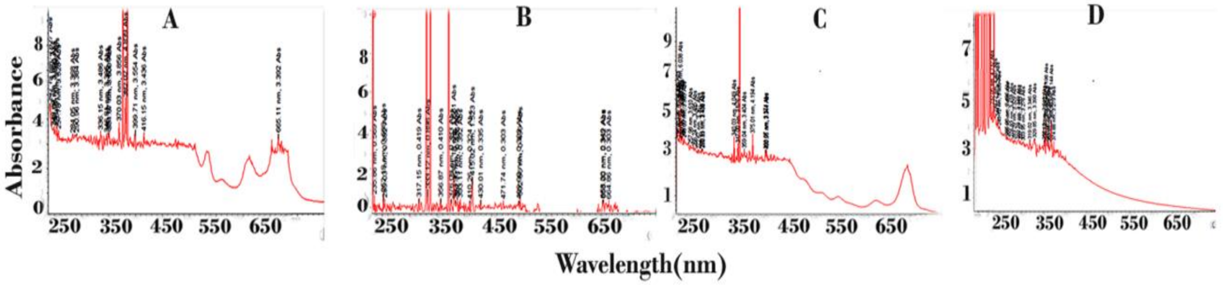

Stem

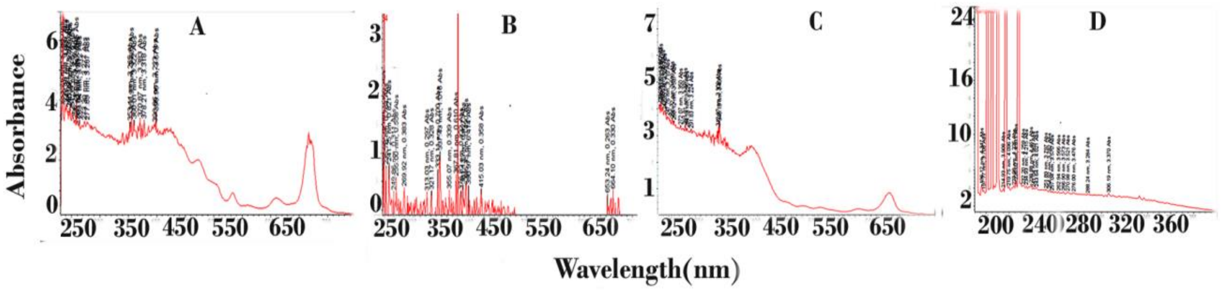

Flower

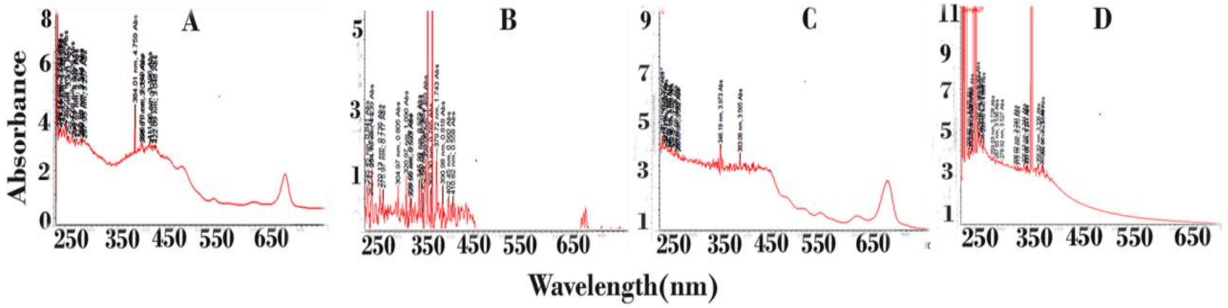

Supplementary Figure 2: Spectrophotometric analysis of the extracts of leaves, stem and flowers of *P. hysterophorus* prepared in different solvents at low temperature using Soxhlet apparatus showing their varying absorption abilities of light in the UV-Visible range. Solvents used were A=Hexane, B= Ethyl acetate, C=Methanol, and D=Aqueous
